# Function of Chemokine (CXC Motif) Ligand 12 in Periodontal Ligament Fibroblasts

**DOI:** 10.1371/journal.pone.0095676

**Published:** 2014-05-07

**Authors:** Yuichi Yashiro, Yoshiaki Nomura, Mikimoto Kanazashi, Koji Noda, Nobuhiro Hanada, Yoshiki Nakamura

**Affiliations:** 1 Department of Orthodontics, School of Dental Medicine, Tsurumi University, Yokohama, Japan; 2 Department of Translational Research, School of Dental Medicine, Tsurumi University, Yokohama, Japan; 3 Department of Periodontology, School of Dental Medicine, Tsurumi University, Yokohama, Japan; The University of Adelaide, Australia

## Abstract

The periodontal ligament (PDL) is one of the connective tissues located between the tooth and bone. It is characterized by rapid turnover. Periodontal ligament fibroblasts (PDLFs) play major roles in the rapid turnover of the PDL. Microarray analysis of human PDLFs (HPDLFs) and human dermal fibroblasts (HDFs) demonstrated markedly high expression of chemokine (CXC motif) ligand 12 (*CXCL12*) in the HPDLFs. CXCL12 plays an important role in the migration of mesenchymal stem cells (MSCs). The function of CXCL12 in the periodontal ligament was investigated in HPDLFs. Expression of *CXCL12* in HPDLFs and HDFs was examined by RT-PCR, qRT-PCR and ELISA. Chemotactic ability of CXCL12 was evaluated in both PDLFs and HDFs by migration assay of MSCs. CXCL12 was also immunohistochemically examined in the PDL *in vivo*. Expression of *CXCL12* in the HPDLFs was much higher than that in HDFs *in vitro*. Migration assay demonstrated that the number of migrated MSCs by HPDLFs was significantly higher than that by HDFs. In addition, the migrated MSCs also expressed *CXCL12* and several genes that are familiar to fibroblasts. CXCL12 was immunohistochemically localized in the fibroblasts in the PDL of rat molars. The results suggest that PDLFs synthesize and secrete CXCL12 protein and that CXCL12 induces migration of MSCs in the PDL in order to maintain rapid turnover of the PDL.

## Introduction

The periodontal ligament (PDL) is one of the connective tissues located between the tooth and bone. It has shock-absorbing properties against mechanical stress [1], and thus, it prevents damage to the tooth and alveolar bone during mastication [2], [3]. In addition, the PDL enables teeth to move via periodontal regeneration during orthodontic treatment [4]–[7].

The PDL is composed of heterogeneous cell populations; fibroblasts, osteoblasts, cementoblasts, osteoclasts, mesenchymal cells, mast cells and phagocytes [8]. Among these, fibroblasts are predominant. PDL is characterized by rapid renewal and repair, high remodeling capacity [9]–[11], and a remarkable capacity for renewal and repair when compared with other connective tissues, such as subcutaneous tissue [12]. PDL fibroblasts (PDLFs) are responsible for these characteristics of the PDL.

The supply of fibroblasts in PDL with these characteristics is controversial [13]–[15]. PDLFs are probably a source of osteoblasts and cementoblasts for remodeling of alveolar bone and cementum [16]–[18]. In addition, PDLFs are suitable cell sources of induced pluripotent stem cells [19]. These reports indicate that PDLFs are multipotent and may be capable of self-replication. On the other hand, mesenchymal progenitor cells that differentiate into fibroblasts are also present in the PDL [20]–[22].

Our preliminary results indicated that expression of chemokine (CXC motif) ligand 12 (CXCL12) in HPDLFs was much higher than that in human dermal fibroblasts (HDFs). The function of CXCL12 is to induce migration of mesenchymal stem cells (MSCs) [23]–[25]. Therefore, the objective of this study was to investigate the function of CXCL12 in the PDL with rapid turnover.

## Materials and Methods

### Ethics Statement

All the experiments were conducted in accordance with the guidelines of the National Institutes of Health, and the Ministry of Education, Culture, Sports, Research or publication ethics, Science and Technology of Japan, and were approved by the Animal Research Committee of Tsurumi University, Kanagawa, Japan. We made every effort to minimize the number of animals used and their suffering.

### Cell Culture

Normal human periodontal ligament fibroblasts (HPDLFs) and human dermal fibroblasts were derived from six different donors respectively; a 16-year-old male (HPDLFs1) (Lonza Biosciences, Basel, Switzerland), a 26-year-old female (HPDLFs2) (Lonza Biosciences), a 17-year-old male (HPDLFs3) (Lonza Biosciences) for HPDLFs and fetal dermis (HDFs1) (KURABOU Co., Ltd., Osaka, Japan), neonatal foreskin (HDFs2) (TOYOBO Co., Ltd., Tokyo, Japan), and 34-year-old abdominal skin (HDFs3) (TOYOBO) for HDFs. Cells were maintained in stromal cell basal medium (SCBM™, Takara Bio Inc., Otsu, Japan) supplemented with growth factors (basic fibroblast growth factor, insulin), 10% FBS and gentamicin/amphotericin-B. Normal human dermal fibroblasts were maintained in Medium106S supplemented with low serum growth supplement (LSGS Life Technologies, Carlsbad, CA). As a negative control, human epithelial cells HeLa were used [19]. The human epithelial cell line HeLa was maintained in DMEM supplemented with 10% FBS and streptomycin (100 µg/ml)/penicillin (100 IU/ml). Human MSCs were also derived from three different donors; UCB408E6E7TERT-33 (MSCs1) was derived from human umbilical cord blood, and UE7T-13 (MSCs2) and UE6E7T-11 (MSCs3) were derived from human bone marrow. These cells were immortalized by transformation with HPV E6, HPV E7 and hTERT, which was purchased from Riken Cell Bank (Ibaraki, Japan). MSCs were maintained in complete medium including serum (PLUSOID-M; GP Biosciences Co., Ltd., Yokohama, Japan) and were cultured in a humidified incubator containing 5% CO_2_ and 95% air at 37°C. Cells at passages three and five were used in this study.

### RNA Extraction and cDNA Synthesis

Total RNAs were extracted from the cell lines described above using Isogen (Nippon Gene Co., Ltd., Toyama, Japan), and 1 µg of total RNA was reverse transcribed to complementary DNA (cDNA) using a PrimeScript II 1st strand cDNA synthesis kit (Takara Bio Inc.) with random hexamers.

### Microarray Analysis

Microarray analysis was performed using a Whole Human Genome 4×44K (Agilent Technologies, Tokyo, Japan) containing approximately 44,000 transcripts. According to the manufacturer’s protocol, total RNAs from HPDLFs and HDFs were labeled with Cy3, respectively, mixed, and hybridized on the microarray. Hybridization data for HPDLFs were compared with data for HDFs.

### RT-PCR

RT-PCR analysis was performed with a Sapphire-Amp™ Fast PCR Master Mix (Takara Bio Inc.) using cDNA derived from 100 ng of total RNA in a 25-µl reaction volume. All reactions were carried out in triplicate. PCR conditions were 94°C for 1 min, followed by 25–40 cycles of denaturation at 98°C for 5 s, annealing of specific primers for 5 s, and extension at 72°C for 5 s. Primer sequences were obtained from published DNA sequences of human genes expressed in periodontal connective tissue or osteogenesis markers [26]–[31]. Primer sequences and annealing temperatures for each reaction are shown in Table 1.

**Table 1:** Primer sequences used in this study.

GenBank accession no.	Symbol	Primer Pairs	Sequence (5′→3′)	Amplification length	*T*m	Riferences
NG_007073	*GAPDH*	sense	acccagaagactgtggatgg	138 bp	50.0	[19]
		antisense	cagtgagcttcccgttcag		49.4	
NG_007400	*COL1A1*	sense	gattgaccccaaccaagg	724 bp	62.8	[28]
		antisense	agtgacgctgtaggtgaagc		62.1	
NM_000088.3	*COL1A1*	sense	cagccgcttcacctacag	72 bp	62.2	[32]
(qRT-PCR)		antisense	aatcactgtcttgccccagc		66.4	
NG_007404	*COL3A1*	sense	cagtattctccactcttgagttcag	555 bp	62.7	[29]
		antisense	ggtgacaaaggtgaaacaggtgaac		63.8	
NG_007404	*COL3A1*	sense	tggcacaacaggaagctgttgaagg	97 bp	73.6	[32]
(qRT-PCR)		antisense	acacatatttggcatggttctggct		69.8	
NM_004334	*BST1*	sense	gcatccatccagtattccaagg	417 bp	66.3	[28]
		antisense	aagccagcaccagaaagagg		65.6	
NG_008076	*GDF5*	sense	aagcgacccagcaagaacc	418 bp	66.5	[28]
		antisense	tcctgacccctctgtgattcc		67.5	
NM_001191322	*GREM1*	sense	ctcaactgccctgaactacagc	748 bp	65.0	[28]
		antisense	tccctttctcactccactatcc		63.2	
NM_006350	*FST*	sense	tgggaatgatggagtcacc	554 bp	63.5	[28]
		antisense	caacaacagcgcagaagc		63.5	
NG_008940	*ALP*	sense	gcacctgccttactaactcc	704 bp	60.3	[28]
		antisense	catgatcacgtcaatgtcc		59.9	
NM_199168.3	*CXCL12*	sense	agtcaggtggtggcttaacag	150 bp	63.0	[30]
		antisense	agaggaggtgaaggcagtgg		65.7	
NG_023430	*PLAP1*	sense	ctgggcctaggaaacaacaa	212 bp	63.8	[31]
		antisense	ttggcactgttggacagaag		63.9	

### Quantification of CXCL12

qRT-PCR analysis was performed with SYBR Premix Ex Taq II Perfect Real Time reagent (Takara Bio Inc.) in a Thermal Cycler Dice Real Time System (Takara Bio Inc.) using a specific primer pair of each cDNA according to the published sequences for *COL1A1* [28], [32] and *COL3A1* [29], [32]. PCR conditions were 95°C for 30 s, 40 cycles of denaturation at 95°C for 5 s and annealing and extension at 60°C for 30 s, followed by dissociation and preparation of a standard denaturation curve. The standard curve for each gene was plotted showing Ct versus the logarithmic value of diluted concentrations of the target cDNA standard. The amount of endogenous mRNA of *CXCL12* and *GAPDH* in these cells was quantified by assumption from the standard curve for each gene, as in our previous study [27]. The mean values and standard deviations were calculated from three independent experiments. Analysis was also performed on other genes.

### Quantification of CXCL12 Protein

Culture medium incubated for 24 h was collected using a pipette, and was centrifuged 1000 rpm at 4°C for 10 min. Supernatants were used for protein analysis. Quantification of CXCL12 in culture supernatants of HPDLFs and HDFs was performed using a commercially available Quantkine ELISA kit (R&D Systems, Minneapolis, MN). CXCL12 concentrations were calculated with reference to a standard curve. All samples were analyzed in triplicate.

### siRNA Transfection

Small interfering RNA targeting *CXCL12* (CXCL12-siRNA) and scramble siRNA as a negative control were transfected into cells with jetPRIME (Polyplus, Illkirch, France) according to the manufacturer’s recommendations. Briefly, HPDLFs were seeded in 24-well plates at a density of 1.5×10^5^ cells per well in 2 ml of Opti-MEM I Reduced-Serum Medium (Life Technologies). HPDLFs were transfected with scrambled siRNA (negative control) or CXCL12-siRNA diluted in transfection buffer at a final concentration of 50 nM. Fifty microliters of transfection medium was added to 24-well plates. After 24 h, transfection medium was replaced with culture medium, and the following day, transfected HPDLFs were used for migration assay and all knockdown studies. CM was also used in the migration assay.

### In Vitro Transwell Chamber Migration Assay

A Falcon cell culture insert system and a companion 24-well Falcon tissue culture plate (Becton Dickinson, Franklin Lakes, NJ) were used for migration assay, which was performed according to the manufacturer’s instructions. Migration assay was performed using MSCs from three donors. Cell cytoplasm of MSCs was stained with DiI Fluorescent Dye (Becton Dickinson) for 30 min at 37°C in the dark, and then 1×10^5^ cells in 200 µl of PLUSOID-M were seeded on the upper surface of the filter membrane in the upper wells. The lower wells were seeded with 1.5×10^5^ HPDLFs from three donors to confirm reactivity to HPDLFs. Chambers were incubated for 24 h at 37°C. Nuclei of migrated cells on the lower surfaces were stained with DAPI and were observed by fluorescence microscopy. The number of migrating cells in each microscopic field (×100) was also counted. There were no significant differences in the number of migrated cells among MSCs from three donors. Therefore, MSCs from one donor were used in subsequent experiments. Lower wells were also seeded with 1.5×10^5^ CXCL12-siRNA transfected HPDLFs, scramble siRNA transfected HPDLFs or HDFs. Human recombinant CXCL12 (250 ng/ml; PeproTech, Rocky Hill, NJ) in 500 µl of PLUSOID-M was used as a positive control, and was added to the medium in the lower well. Non-migrating cells on the upper surfaces of the filter membrane were removed with a swab. Nuclei of migrated cells on the lower surfaces were stained with DAPI and observed by fluorescence microscopy. The number of migrating cells in each microscopic field (×100) was also counted. Medium without cells served as a negative control. Experiments were performed in triplicate. For blocking of CXCR4, CXCR4 neutralizing antibody (100 µg/ml; Abcam, Cambridge, UK) or IgG antibody (negative control, 100µg/ml; Abcam) were added to upper wells [33]. In addition, to block CXCR4 receptors, MSCs were first incubated in 1.0 µg/ml AMD3100 (Sigma, St. Louis, MO) for 30 min at room temperature, washed in PBS and then resuspended in PLUSOID-M [34].

### Exposure of MSCs to HPDLFs CM

HPDLFs, HDFs, CXCL12-downregulated HPDLFs and scramble siRNA transfected HPDLFs were incubated in a 6-well plate (2×10^5^ cells/ml) with PLUSOID-M culture medium. After 24 h, culture medium was collected using a pipette, and was centrifuged 1000 rpm at 4°C for 10 min. Supernatants from these cells were used for CM. MSCs (2×10^5^ cells/ml) were exposed to CM under the previously described conditions, and medium only was used for 24 h as a negative control. MSCs were analyzed by quantitative RT-PCR.

### Immunohistochemistry

After perfusion with 4.0% paraformaldehyde (0.1 M PBS buffer, pH 7.4, room temperature) for 5 min, upper first molars including its periodontal ligament (PDL) were excised from the maxilla of rats. Specimens were decalcified with 0.3 M EDTA-Na2 (7% sucrose, 4°C) for 2 weeks, and were washed overnight in 0.1 M PBS buffer. After dehydration in graded ethanol, specimens were embedded in paraffin, and were cut serially with a microtome (sliding type, Sakura Seiki, Tokyo) at 6–7 µm thickness. Some deparaffinized sections were stained with HE. Other sections were incubated in a 1:100 dilution of anti-CXCL12 (host: rabbit, polyclonal type, SC-109854; Santa Cruz Biotechnology, Santa Cruz, CA) as primary antibody in a conventional procedures, followed incubation in peroxidase-labeled anti-rabbit IgG (MP-7500; ImmPRESS Reagent, Vector Lab., Inc., Peterborough, UK) as secondary antibody. After incubation, sections were immunostained with DAB and counterstained with hematoxylin. The sections incubated without primary antibody were used as a negative control.

### Statistical Analysis

All data are expressed as means and standard deviation from three independent experiments. Differences among independent groups were analyzed by one-way analysis of variance (ANOVA) followed by Bonferroni’s multiple comparison using statistical software (SPSS Statistics ver. 19.0; IBM Japan, Tokyo, Japan). P-values of less than 0.05 were considered to be statistically significant.

## Results

### Microarray Analysis of HPDLFs and HDFs

Microarray analysis demonstrated that 3,807 genes in HPDLFs were expressed at levels more than 2-fold higher than in HDFs. Array data was deposited at the National Center for Biotechnology Information (NCBI) under the accession number GSE52162. The most remarkable result was observed in the expression of *CXCL12* (209-fold), which is a migration-associated cytokine (Table 2). The expression of *COL1A1* (26.1-fold) and *COL3A1* (41.8-fold) in HPDLFs was higher than in HDFs. Expression of specific markers of connective tissues such as *BST1* (15.3-fold), *GDF5* (10.3-fold), *Gremlin* (8.8-fold) and *Follistatin* (*FST*) (6.6-fold) were also higher in HPDLFs than in HDFs.

**Table 2:** Gene expression of HPDLFs compared with HDFs by microarray analysis.

GenBank accession no.	Symbol	Description	Fold change (PDLFs *vs.* HDFs)
NM_199168	*CXCL12*	*Homo sapiens chemokine (C-X-C motif) ligand 12*	208.8
NM_000090	*COL3A1*	*Homo sapiens collagen, type III, alpha 1*	41.8
NM_000088	*COL1A1*	*Homo sapiens collagen, type I, alpha 1*	26.1
NM_004334	*BST1*	*Homo sapiens bone marrow stromal cell antigen 1*	15.3
NM_000557	*GDF5*	*growth differentiation factor 5*	10.3
NM_013372	*Gremlin*	*Homo sapiens gremlin 1, cysteine knot superfamily*	8.8
NM_013409	*Follistatin*	*Homo sapiens follistatin (FST), transcript variant FST344*	6.6

### Confirmation of Gene Expression Levels by RT-PCR in HPDLFs, HDFs and HeLa Cell Lines

**Figure 1:**
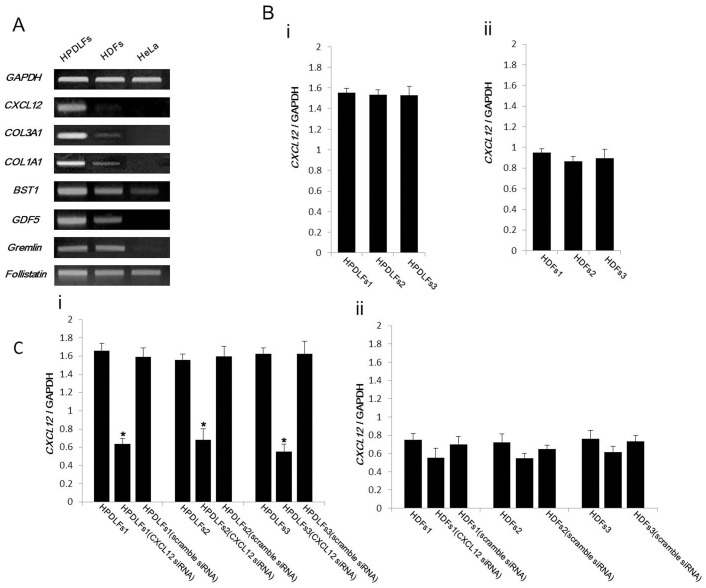
Results of RT-PCR and qRT-PCR. (A) RT-PCR of expression of *CXCL12* and connective tissue specific markers. *CXCL12* in HPDLFs showed distinctly higher expression when compared to HDFs and HeLa cells. (Bi, ii) qRT-PCR of expression of *CXCL12* in HPDLFs and HDFs from three donors for each population. There were no significant differences in the expression of *CXCL12* in the HPDLFs among three donors (HPDLFs1, HPDLFs2 and HPDLFs3) and also in the HDFs among three donors (HDFs1, HDFs2 and HDFs3) (P>0.05). (**C**): Effects of CXCL12-siRNA in HPDLFs from three donors and in HDFs from three donors. Significant down-regulation was commonly observed in HPDLFs among three donors (i). However, expression of CXCL12 was not influenced by CXCL12 siRNA in HDFs from three donors (ii).

In order to confirm the results of microarray analysis, RT-PCR was performed for *CXCL12* in HPDLFs, HDFs and HeLa cells (Fig. 1A). *CXCL12* in HPDLFs showed distinctly higher expression when compared with HDFs and HeLa cells. Expression of *COL3A1* and *COL1A1* in HPDLFs was higher than in HDFs. On the other hand, *Gremlin*, *BST1*, *FST* and *GDF5* were expressed in both HPDLFs and HDFs, but no clear differences in expression were observed between the two types of fibroblast. In HeLa cells as a negative control, only *BST1* and *FST* were detected, which is consistent with our previous report [19].

### Examination of Gene Expression of CXCL12 in HPDLFs and HDFs from three Donors for Each Population by qRT-PCR

Expression of *CXCL12* in HPDLFs derived from three donors was examined by qRT-PCR in order to confirm common characteristics in HPDLFs. This confirmation was also performed in HDFs from three donors (Fig. 1B). There were no statistically significant differences in the expression of *CXCL12* among the fibroblasts from the three donors of HPDLFs (Fig. 1Bi) or among fibroblasts from the three donors of HDFs (Fig. 1Bii).

The effects of CXCL-siRNA on HPDLFs and HDFs were also examined three donors for each population (Fig. 1C). The results showed significant down-regulation in the expression of CXCL12 in HPDLFs from three donors (Fig. 1Ci). But the expression of CXCL12 in HDFs was not influenced by CXCL12-si-RNA in three donors (Fig. 1Cii). Therefore, HPDLFs from one donor of HPDLFs (HPDLFs1) and HDFs from one donor were used in subsequent analysis.

### Confirmation of Gene Expression and Protein Expression Levels of CXCL12 in HPDLFs and HDFs by qRT-PCR and ELISA for CXCL12

**Figure 2:**
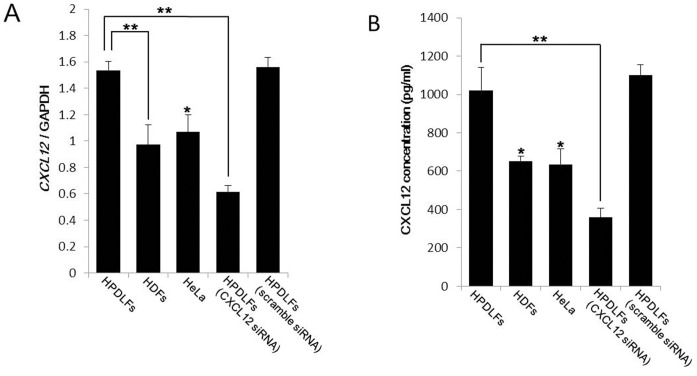
qRT-PCR and ELISA of expression of CXCL12 in HPDLFs, HDFs, HeLa and siRNA transfected HPDLFs. (A) qRT-PCR of CXCL12 in HPDLFs and HDFs. Expression of *CXCL12* in HPDLFs was significantly higher than in HDFs. *CXCL12*-siRNA transfected HPDLFs showed significantly lower expression of *CXCL12*. (B) ELISA of CXCL12 in HPDLFs and HDFs. CXCL12 protein secreted by HPDLFs, HDFs and HeLa cells in culture medium was measured by ELISA. Expression of CXCL12 in the supernatant from HPDLFs was significantly higher than that from HDFs and HeLa cells at the protein level. CXCL12-siRNA-transfected HPDLFs showed significantly lower expression, when compared with expression of CXCL12 in the HPDLFs. * and ** denote p values less than 0.05 and 0.01, respectively, by Bonferroni’s multiple comparison.

qRT-PCR demonstrated that expression of *CXCL12* in HPDLFs was significantly higher than in HDFs (Fig. 2A). Expression of *CXCL12* in HDFs was as low as in HeLa cells. The expression of CXCL12 protein in the conditioned medium (CM) was examined by ELISA (Fig. 2B). CXCL12 in CM from HPDLFs was significantly higher than that in CM from HDFs and HeLa cells. These results are consistent with the results of RT-PCR. The effects of CXCL12-siRNA caused a marked reduction in the expression of CXCL12 in both mRNA and protein levels. On the other hand, HPDLFs with transfection of scrambled siRNA did not show significant differences in expression of CXCL12 when compared with parental HPDLFs. The effects of CXCL12-siRNA transfection on mRNA levels were also examined from 0 to 72 h. The effects continued until 72 h.

### Migration Assay of MSCs by HPDLFs

**Figure 3:**
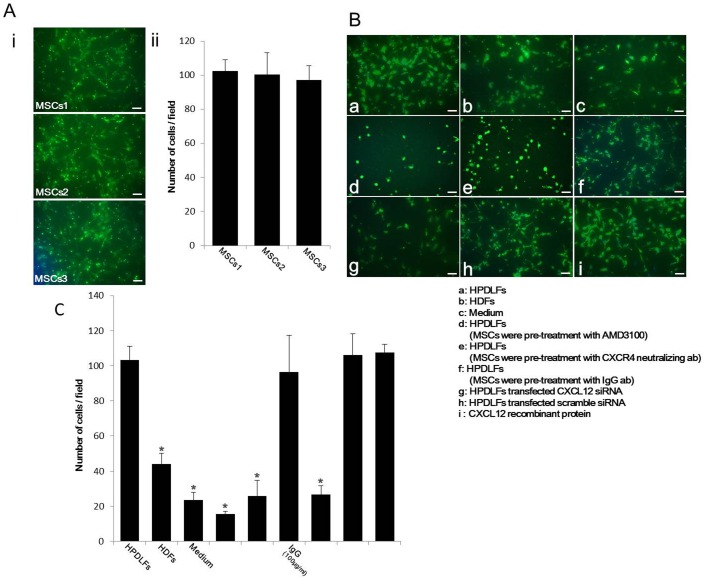
MSC migration assay. (Ai) Reactivity of MSCs from three donors to HPDLFs. Fluorescent microscopy showed no differences in the number of migrated cells of MSCs among the three donors. (Aii) Statistical analysis of migrated MSCs. There were no significant differences in the number of migrated cells of MSCs among the three donors. (B) Migration assay of MSCs. Fluorescent microscopy demonstrated that migrated MSCs by HPDLFs (**a**) were much more abundant than those by HDFs (**b**) and medium alone (c)**. a**: HPDLFs **b**: HDFs **c**: Medium only **d**: HPDLFs (MSCs were pre-treated with 5 µg/ml AMD3100) **e**: HPDLFs **(**MSCs were pre-treated with 100-µg/ml CXCR4-neutralizing antibody) **f**: HPDLFs (MSCs were pre-treated with 100-µg/ml IgG antibody) **g**: HPDLFs (transfected with CXCL12-siRNA) **h**: HPDLFs (transfected with scramble siRNA) **i**: recombinant CXCL12. *Bars, 100 µm*. Migrating MSCs by HPDLFs (**a**) were much more abundant than those by HDFs (**b**) and medium alone (c). (C) Statistical analysis of migrated MSCs. Number of migrating MSCs from HPDLFs (106.7±2.51 cells per field, P<0.01) was significantly higher than those from HDFs (39.3±2.30 cells per field, P<0.01), medium only (23.6±4.16 cells per field, P<0.01) and CXCL12-siRNA-transfected HPDLFs (25.3±0.57 cells per field, P<0.01). When MSCs were pre-treated with AMD3100 (15.6±1.52 cells per field, P<0.01) or CXCR4 antibody (25.6±9.07 cells per field, P<0.01), the number of migrated cells was significantly lower. *P<0.01 number of migrated MSCs by HPDLFs.

Prior to comparison among the results, MSCs from three different donors were examined in this assay to confirm the reactivity to HPDLFs (Fig. 3Ai). There were no statistically significant differences in the number of migrated cells among MSCs from three donors (Fig. 3Aii). MSCs from one donor were used in subsequent assays. The chemotactic activity of CXCL12 in HPDLFs, HDFs and CXCL12-siRNA-transfected HPDLFs was examined by transwell chamber migration assay. Fluorescent microscopy clearly demonstrated the results of the assay (Figs. 3Ba–i). MSCs passing through the filter membrane were observed on the lower surface of the membrane. Migrated MSCs by HPDLFs were much more abundant (Fig. 3Ba) on the lower surface of the filter membrane than those by HDFs (Fig. 3Bb) and medium only (Fig. 3Bc). The results of migration using inhibitor of CXCR4 (Fig. 3Bd) and CXCR4 antibody (Fig. 3Be) showed similar migration as with the results of medium alone. This was also observed in the migration of MSCs, using CXCL12-siRNA (Fig. 3Bg). On the other hand, migrated MSCs by scramble siRNA-transfected HPDLFs (Fig. 3Bh), IgG antibody (Fig. 3Bf) and recombinant CXCL12 (Fig. 3Bi) showed the similar results as HPDLFs.

The number of migrated MSCs was also counted at 24 h after initiation of the assay (Fig. 3C). The number of migrated MSCs by HPDLFs was significantly higher than that by HDFs. Recombinant CXCL12 also induced similar migration as with HPDLFs. The number of migrating cells on by medium alone, CXCR4 inhibitor and CXCR4 antibody was significantly lower than that by HPDLFs.

### Examination of Gene Expression in Migrated MSCs by RT-PCR and qRT-PCR

**Figure 4:**
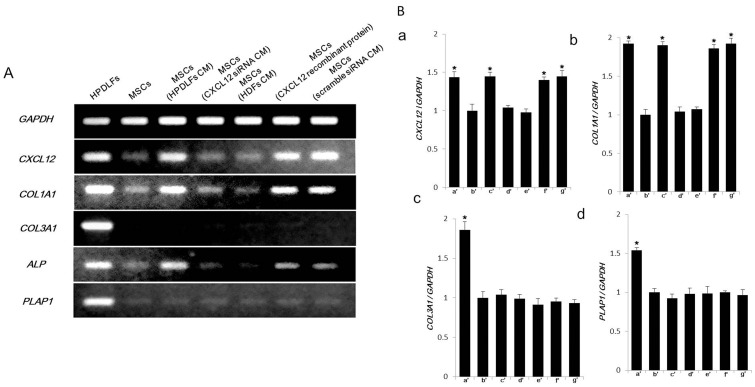
RT-PCR and qRT-PCR of gene expression in MSCs exposed to CM from HPDLFs and CM from CXCL12 down-regulated HPDLFs. (A) RT-PCR of gene expression in MSCs. Expression of *CXCL12* was up-regulated in MSCs exposed to CM from HPDLFs at 24 h after incubation. *COL1A1* and *Alkaline phosphatase* were also up-regulated in MSCs. (B) qRT-PCR of gene expression in MSCs. *CXCL12* (a–c’) and *COL1A1* (b–c’) expressions were significantly up-regulated in MSCs exposed to CM from HPDLFs, as compared with gene expression of MSCs in control medium (a–b’ and b–b’). However, up-regulation was not observed in the expression of *COL3A1* (c–b’) and *PLAP1* (d–b’). MSCs exposed to CM from CXCL12-siRNA transfected HPDLFs (a–d’ and b–d’) or MSCs (a–b’ and b–b’) alone did not show up-regulation in expression of *CXCL12* and *COL1A1*. There were no significant differences in the expression of COL3A1 (c–b’–g’) and PLAP1 (d–b’–g’). **a’**: HPDLFs **b’**: MSCs (Medium only (control)) **c’**: MSCs (HPDLFs CM) **d’**: MSCs (CXCL12-siRNA transfected HPDLFs CM) **e’**: MSCs (HDFs CM) **f’**: MSCs (medium contained recombinant CXCL12) **g’**: MSCs (scramble siRNA-transfected HPDLFs CM). All samples compared to MSCs cultured in normal culture medium (b’). *denotes p values less than 0.01.

The influence of CM from HPDLFs on MSCs was examined by RT-PCR (Fig. 4A). Results were compared to those with MSCs cultured in normal culture medium. Expression of *CXCL12* was up-regulated in the MSCs exposed to CM from HPDLFs at 24 h after incubation. *COL1A1* and Alkaline phosphatase were up-regulated in MSCs. Up-regulation was also observed in medium containing recombinant CXCL12. These results were supported by qRT-PCR (Figs. 4Ba–d). *CXCL12* and *COL1A1* expression was also significantly up-regulated in MSCs. Up-regulation was not observed in the expression of *COL3A1* and *PLAP1*. MSCs exposed to CM from CXCL12-siRNA HPDLFs or MSCs alone did not show up-regulation in expression of *CXCL12* and *COL1A1*.

### Immunohistochemistry for CXCL12 in PDL in vivo

**Figure 5:**
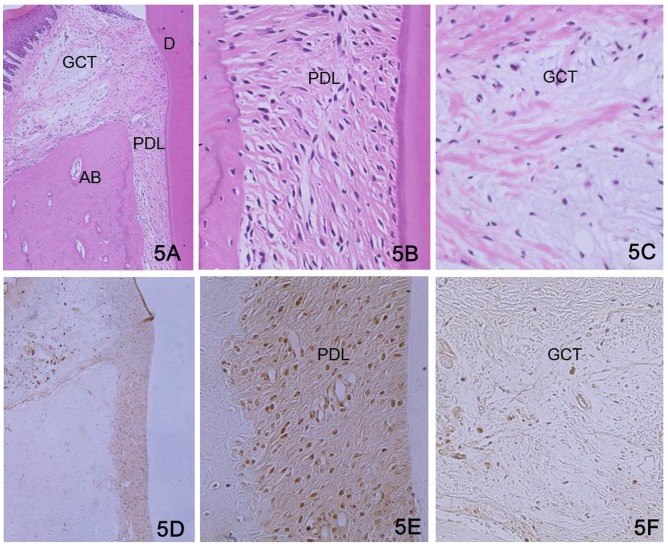
Immunohistochemistry of CXCL12 in PDL of rat molars. (A) Histology of periodontal tissues. Periodontal tissue consists of PDL, alveolar bone, cementum and gingiva. (B) Histology of periodontal ligament. Fibroblasts were observed throughout the PDL and scattered along the periodontal fibers. (C) Histology of gingival connective tissue. Fibroblasts were also scattered and some capillaries were present in the gingival connective tissue. (D) Localization of CXCL12 in periodontal tissue. CXCL12 was predominantly localized in fibroblasts in PDL. (E) Localization of CXCL12 in PDL. CXCL12 was localized in the most fibroblasts. (F) Localization of CXCL12 in gingival connective tissues. CXCL12 was not observed in most fibroblasts, and was detected only in the cells around the capillaries. G: Control section of immunohistochemistry. CXCL12 was not detected in the periodontal ligament. AB: Alveolar bone, PDL: Periodontal ligament, GCT: Gingival connective tissue, D: Dentine. A: HE stain×50, B and C HE stain×200, D: Immunohistochemistry×50, E and F: Immunohistochemistry×200, G: Control section of immunohistochemistry×200.

Periodontal tissue consists of PDL, alveolar bone, cementum and gingiva. Fibroblasts were observed throughout the PDL and scattered along the periodontal fibers (Fig. 5A,B). In gingiva, fibroblasts were scattered and some capillaries were present in the gingival connective tissue (Fig. 5C). CXCL12 was predominantly localized in the fibroblasts in the PDL (Fig. 5 D, E). However, CXCL12 was not observed in most of the fibroblasts in the gingival connective tissue (Fig. 5D, F), and was only detected in cells around the capillaries.

## Discussion

HPDLFs and HDFs used in this study were commercially available cell lines. It is important to confirm the characteristics of HPDLFs and HDFs. Both fibroblasts showed a spindle-like shape when grown in culture dishes. These fibroblasts clearly showed expression of several connective tissue-specific markers on RT-PCR [27]–[32], which were different from those in the human epithelial cell line Hela (negative control), except for *BST1* and *follistatin*. The HPDLFs and HDFs used in this study had the characteristics of connective tissue fibroblasts. In this study, three cell lines of HPDL fibroblasts, and HD fibroblasts were used to examine the differences in expression of CXCL12, and consequently, no differences were observed. This indicates that CXCL12 is commonly expressed in HPDLFs, and that its expression is significantly higher than in HDFs. In addition, MSCs from three donors were also examined to confirm the reactivity to the chemotactic activity of HPDLFs, and there were no significant differences in the number of migrated cells among the three donors.

RT-PCR analysis showed that both HPDLFs and HDFs expressed *COL1A1* and *COL3A1*, and that HeLa cells did not express these genes. This indicates that both HPDLFs and HDFs in this study have the characteristics of fibroblasts. However, it appears that the expression of *COL1A1* and *COL3A1* in HPDLFs was much stronger than in HDFs. In general, the PDL shows rapid turnover and PDLFs play distinct functional roles in the regeneration and repair of the PDL [35], [36] and the collagen synthesis is one of the primary functions of PDLFs [37], [38], HPDLFs have fundamentally high activity in the formation of collagen fibers [39], [40].

Microarray analysis of the whole genome revealed distinctly high expression of *CXCL12* in HPDLFs when compared to that in HDFs. qRT-PCR demonstrated significant upregulation of *CXCL12* in HPDLFs. These results indicate that *CXCL12* is upregulated not only in the cells under pathological conditions such as inflammation and tumors [41], [42], but also in normal HPDLFs. The high expression of *CXCL12* in HPDLFs was also confirmed at the protein level by ELISA. Therefore, HPDLFs synthesize CXCL12 protein and secrete it extracellularly. In addition, the immunohistochemical findings showed that CXCL12 is located in fibroblasts in the PDL.

The function of CXCL12 in HPDLFs was investigated by migration assay. CXCL12 is involved in cell migration [33], [43]–[45]. Our migration assay demonstrated that HPDLFs induced migration of a significantly higher number of MSCs than did HDFs. HPDLFs in which CXCL12 was downregulated by siRNA induced low migration, which was equivalent to the migration induced by HDFs. Furthermore, recombinant CXCL12 induced migration of MSCs, which is similar to that of HPDLFs. These results precisely define the function of CXCL12 in HPDLFs. CXCL12 plays a critical role in the supply of fibroblasts that are responsible for rapid turnover. This migration may result from the increased mobility of plasma membranes in response to CXCL12, which induces cytoskeletal arrangement, pseudopodia formation [46] and consequently MSC pass through the filter membrane. MSCs usually circulate in the blood vessels [47]–[49] and PDL is rich in blood vessels and shows rapid turnover, when compared with other connective tissues [50], [51]. Thus, we postulated that CXCL12 secreted from PDLFs induces migration of MSCs from the blood to the site of rapid renewal and repair. Furthermore, CXCL12 may be a specific marker that distinguishes PDLFs from other fibroblasts.

On the other hand, the treatment with CXCL12 siRNA did not affect the expression of CXCL12 in HDFs population. This might be because the expression level of CXCL12 is intrinsically low in HDFs, which is closely related to low turnover in dermal connective tissue, as compared with that in PDL.

This study also noted an interesting finding regarding MSCs. MSCs incubated in CM from HPDLFs showed some changes in gene expression patterns. The CM from HPDLFs induced up-regulation of *CXCL12*, *COL1A1* and *ALP* in MSCs. However, MSCs incubated in CM from HPDLFs in which CXCL12 was down-regulated by CXCL12-siRNA, did not show the same changes and instead, showed similar results as control MSCs. The up-regulation of CXCL12 in MSCs may induce new migration of MSCs via synthesis and secretion of CXCL12 during migration, which also contributes to the rapid renewal and repair of the PDL. Furthermore, the up-regulation of *COL1A1* and *ALP* in MSCs suggests the possible differentiation of MSCs into fibroblast-like cells during migration. This might be due to the activation of MAP kinase by CXCL12 [52], [53], which induces up-regulation of *COL1A1* [54]–[56] and *ALP* [57], [58]. In addition, COL1A1 and ALP are requisite factors of PDL fibroblasts [59]. Collagen type I is a primary component of periodontal fibers and ALP is located in collagen fibers and fibrils, which are different characters from other connective tissues. Therefore, *COL1A1* and *ALP* might be given priority over the expression of other genes. In summary, the results in this study suggest that PDLFs synthesize and secrete CXCL12. CXCL12 from PDLFs induces migration of MSCs to supply new fibroblasts in the PDL with rapid turnover.
